# Alkali Activation of Metakaolin and Wollastonite: Reducing Sodium Hydroxide Use and Enhancing Gel Formation through Carbonation

**DOI:** 10.3390/ma17194910

**Published:** 2024-10-08

**Authors:** Veronica Viola, Prince Allah, Priyadharshini Perumal, Michelina Catauro

**Affiliations:** 1 Department of Engineering, University of Campania “Luigi Vanvitelli”, Via Roma 29, 81031 Aversa, Italy; veronica.viola@unicampania.it; 2 Fibre and Particle Engineering Research Unit, Faculty of Technology, 90014 Oulu, Finland; prince.allah@oulu.fi

**Keywords:** alkali-activated materials, metakaolin, wollastonite, industrial waste, recycling

## Abstract

Alkali activated materials (AAMs) offer significant advantages over traditional materials like Portland cement, but require the use of strong alkaline solutions, which can have negative environmental impacts. This study investigates the synthesis of AAMs using metakaolin and wollastonite, aiming to reduce environmental impact by eliminating sodium silicate and using only sodium hydroxide as an activator. The hypothesis is that wollastonite can provide the necessary silicon for the reaction, with calcium from wollastonite potentially balancing the negative charges usually countered by sodium in the alkaline solution. This study compares raw and carbonated wollastonite (AAM-W and AAM-CW) systems, with raw materials carefully characterized and binding networks analyzed using TGA, FT-IR, and XRD. The results show that while wollastonite can reduce the amount of sodium hydroxide needed, this reduction cannot exceed 50%, as higher substitution levels lead to an insufficiently alkaline environment for the reactions. The carbonation of wollastonite enhances the availability of silicon and calcium, promoting the formation of both N-A-S-H and C-A-S-H gels.

## 1. Introduction

Alkali-activated materials (AAMs) are a class of cementitious materials that can be used as substitutes for traditional Portland cement [1]. These materials are considered eco-friendly, because for their production, industrial by-products are usually used [2,3]. This not only reduces waste but also minimizes the need for energy-intensive processes involved in traditional cement production. Notably, the carbon footprint of AAMs, particularly geopolymers, is substantially lower, with some estimates suggesting a reduction of up to 80–90% in CO_2_ emissions compared to traditional cement production methods [4,5,6]. AAMs can be divided into three groups according to their characteristics. The first group, known as the low calcium system or geopolymers, is characterized by its high content of low-calcium supplementary cementitious materials (SCMs), such as class F fly ash [7]. These SCMs have abundant silicon and aluminum ions, acting as primary reactive binding agents in the geopolymerization process. The interaction of SCMs with alkali hydroxides (NaOH or KOH) leads to the formation of a three-dimensional network of aluminosilicates, referred to as a geopolymer gel [8,9]. In contrast, the high calcium system features a higher proportion of calcium-content SCMs, like slag or calcined clay. Both silicon and calcium ions actively participate in the formation of the binding phase. The reaction between Si and Ca ions results in the creation of a calcium aluminum silicate hydrate (C-A-S-H) network [10,11]. The third category, hybrid systems, involves the combination of ordinary Portland cement (OPC) with high-volume SCMs. In this system, OPC initiates the reaction with water, producing calcium hydroxide (Ca(OH)_2_). This serves as a source of calcium ions for the subsequent geopolymerization reaction with SCMs. The synergy between OPC and AAMs in hybrid systems offers advantages such as high strength and durability, while simultaneously contributing to a reduced environmental impact through the incorporation of industrial by-products [12,13].

Alkali activation involves a complex chemical process wherein precursors, primarily alumino-silicate materials, interact and dissolve in activators [4]. The most studied precursors used are ground granulated blast-furnace slag, rice husk ash, silica fume, fly ash, metakaolin, bottom ash, corn cob ash, corundum, and sugarcane bagasse ash [14,15,16,17,18,19,20,21,22,23]. Some unreactive materials are also incorporated in alkali-activated material synthesis as fillers, reducing the need for landfilling [11,12]. Using waste materials in alkali activation has presented opportunities to study how mineral wastes can be incorporated into AAMs.

For example, it is determined that the use of quarry dust in the alkali activation process can generate significant cost benefits for the stone industry while reducing its environmental impacts [24]. However, most of the mineral waste products have so far been used as fillers because of their high crystallinity [25,26]. Calcium-bearing mineral wastes, however, can act as a source of calcium that contributes to the formation of C-A-S-H gel, C-(N)-A-S-H, and CSH [27].

This study aims to investigate the synthesis of AAMs through the partial substitution of metakaolin with wollastonite, a byproduct characterized by its predominant composition of calcium and silicon. To optimize the eco-friendliness of the process, the elimination of sodium silicate is proposed, and only sodium hydroxide is used as the activator. The hypothesis is that wollastonite can supply the necessary silicon for the reaction. Furthermore, it is assumed that the negative charges conventionally counterbalanced by sodium in sodium hydroxide and sodium silicate may be equilibrated through the sodium in sodium hydroxide and the calcium in wollastonite.

However, the crystalline nature of wollastonite could pose a potential challenge, potentially impeding the release of its constituents. To address this issue, two distinct systems have been made:

- One involving the combination of metakaolin, wollastonite, and sodium hydroxide (AAM-W).
- Another incorporating metakaolin, wollastonite subjected to a carbonation process, and sodium hydroxide (AAM-CW).

The carbonation process applied to wollastonite, beyond its benefits in carbon dioxide sequestration, holds the potential to induce an amorphous state in the material [28,29]. This could enhance the availability of silicon within the structure, promoting increased reactivity in the formation process of AAMs.

## 2. Materials and Methods

### 2.1. Raw Materials

The precursors and activators employed in this study were metakaolin (MK), wollastonite (W), carbonated wollastonite (CW), and sodium hydroxide. A commercial MK was used (MetaMax, BASF), while W and sodium hydroxide were purchased from Kerasil Oy, Finland, and Sigma Aldrich.

For the carbonation of W, the powder was placed in a tray, resulting in a thin, evenly distributed layer. The carbonation chamber was configured to maintain precise conditions: a temperature of 25 °C, a relative humidity of 55%, and a CO_2_ concentration of 20% [30].

### 2.2. Experimental Setup

**Figure 1:**
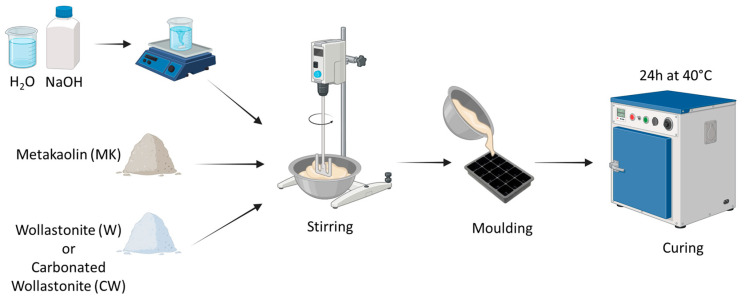
Flowchart of the synthesis process.

The procedure employed for synthesizing the samples is illustrated in Figure 1. Alkaline solutions were prepared by gradually mixing sodium hydroxide pellets with deionized water. These solutions were stirred for 24 h to ensure complete dissolution of the pellets before being used in the synthesis. Specific amounts of metakaolin and wollastonite/carbonated wollastonite were weighed and placed in a mixer to produce the samples. The alkaline solution was then added to the powders to form a paste-like consistency. The mixer was set to a moderate speed for approximately 2–3 min for each synthesis. The mixture was subsequently placed into moulds and covered with transparent film to prevent rapid water evaporation. The moulds were then placed in an oven at 40 °C for 24 h. After 24 h, the samples were removed from the oven and allowed to cool at room temperature. Details regarding the composition of each sample, along with their corresponding labels, are provided in Table 1.

### 2.3. Testing Procedures

W, CW, MK, and samples underwent a comprehensive characterization to evaluate their physical and chemical properties.

#### 2.3.1. PSD

Particle size distribution analysis of W and MK was performed using laser diffraction with a Beckman Coulter LS 13320 instrument (Brea, CA, USA). Isopropanol served as the dispersion medium during the measurement process. The analysis focused on determining the D_10_, D_50_, and D_90_ parameters, which provide insights into the physical characteristics of the material [31]. The span, a measure of the distribution’s breadth, was calculated using the Formula (1) [32]:

(1) $$s=\frac{D_{90}-D_{10}}{D_{50}}$$

#### 2.3.2. XRF and XRD

Elemental composition analysis of W was performed using X-ray fluorescence (XRF, Axios mAX; Malvern PANalytical, Almelo, The Netherlands). The mineralogical composition of both MK and W, as well as the samples, underwent examination through X-ray diffraction (XRD) using a Rigaku SmartLab 9 kW instrument manufactured by Applied Rigaku Technologies in Austin, TX, USA. The instrument employed Co Kα radiation with specific wavelengths (Kα1 = 1.78892 Å; Kα2 = 1.79278 Å; Kα1/Kα2 = 1). Scanning was conducted at a rate of 3°/min within the 5° to 80° (2θ) range, with a step size of 0.02°. Phase identification was accomplished utilizing the X’pert HighScore Plus analytical v.5 software, which accessed the ICDD PDF-5 database.

#### 2.3.3. FT-IR

FT-IR spectroscopy was utilised to assess the carbonation process of the raw materials and to evaluate bond formation in the samples. For this analysis, a Shimadzu FT-IR Prestige21 system (Milan, Italy), equipped with a deuterated triglycine sulphate detector and potassium bromide windows (DTGS KBr), was employed. The measurements were conducted at a resolution of 2 cm^−¹^ with 60 scans collected across the spectral range of 400–2000 cm^−¹^. The analysis involved preparing KBr disks by mixing 2.00 mg of powdered samples with 198.00 mg of KBr. The resulting FT-IR spectra were processed using IR Solution software (version 1.60, Shimadzu, Milan, Italy) and Origin software (version 2022b, OriginLab Corporation, Northampton, MA, USA).

#### 2.3.4. TGA

The TGA/DTG analysis was conducted using a TA Instruments model SDT 650 (New Castle, DE, USA). Exactly 10.00 mg of each of the samples was weighed and positioned in the machine. The temperature range for the analysis spanned from 50 to 1000 °C, with a heating rate of 5 °C/min, all conducted under a nitrogen (N_2_) atmosphere.

## 3. Results and Discussion

### 3.1. Raw Material Characterization

**Figure 2:**
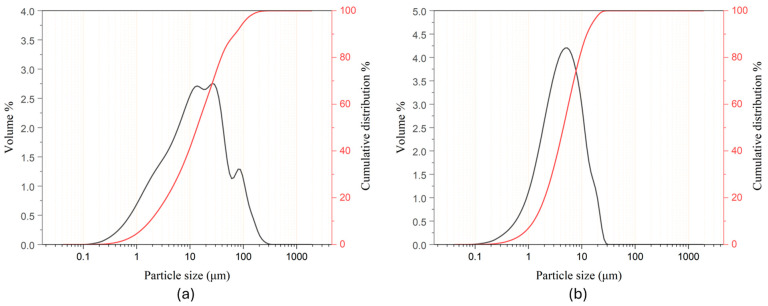
Particle size distribution of wollastonite (**a**) and metakaolin (**b**).

Before sample preparation, the raw materials, namely MK, W, and CW, were carefully analyzed. Figure 2 illustrates the particle size distribution (PSD) of W and MK.

The analysis of W showed particle diameters ranging from 0.04 µm to 350 µm. However, most particles were on the smaller side, as indicated by the D_10_, D_50_, and D_90_ values, which were 1.70 µm, 12.90 µm, and 66.41 µm, respectively. Additionally, the span value, which reflects the homogeneity of the particle sizes, was calculated to be 5.01, suggesting a relatively broad distribution. Smaller particles have a larger surface area, allowing them to react more effectively with the alkaline activator. For W, this increased surface area enhanced the availability of calcium and silicon for reactions, promoting the formation of C-A-S-H gel.

Regarding the MK, particle diameters ranged from 0.04 µm to 19 µm. In this case, the PSD indicated a more homogeneous powder, with D_10_, D_50_, and D_90_ values of 1.20 µm, 4.30 µm and 11.90 µm, respectively, and a span value of 2.49. Similar to W, such small particle sizes, along with their uniformity, ensure the suitability of MK as a precursor for forming AAMs, increasing reactivity and promoting the formation of N-A-S-H gel [33,34].

**Figure 3:**
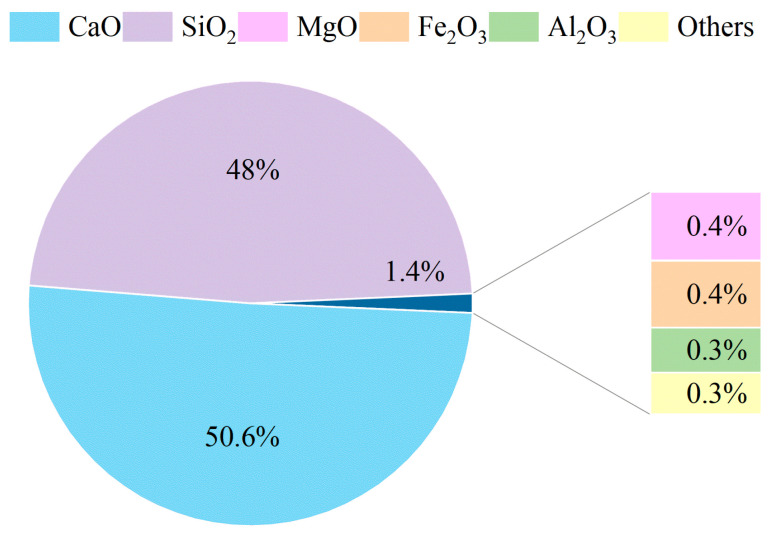
XRF data for wollastonite. “Others” category includes oxides with percentages lower than 0.07%.

The suitability of the raw materials analyzed for the purposes of this study is also evident from the XRF analyses. The composition of the metakaolin (MK) has already been reported in [35], where the same type of MK was found to contain 53.0 wt% SiO_2_ and 44.5 wt% Al_2_O_3_. The XRF analysis of wollastonite (W) is shown in Figure 3. This material was mainly composed of 50.6 wt% CaO and 48.0 wt% SiO_2_.

The high content of SiO_2_ and Al_2_O_3_ in these materials, along with the presence of NaOH solution, is expected to contribute to the formation of N-A-S-H (sodium aluminosilicate hydrate) gel. On the other hand, the combined content of SiO_2_, Al_2_O_3_, and CaO in alkaline environments contributes to the formation of C-A-S-H (calcium aluminosilicate hydrate). The presence of CaO and SiO_2_, in the alkaline environment generated by NaOH solution, can also lead to the formation of C-S-H (calcium silicate hydrate); however, this is less likely to happen since, as reported by [36], the high alkaline pH is more favourable for NASH formation compared to C-S-H formation, which usually is stable at pH 11.

N-A-S-H gel is the primary binding phase in geopolymer systems, while C-A-S-H and C-S-H are typical phases of cementitious binders. The simultaneous formation of these phases implies that the composite material developed through alkali activation could possess properties from both geopolymer and traditional cement chemistry.

**Figure 4:**
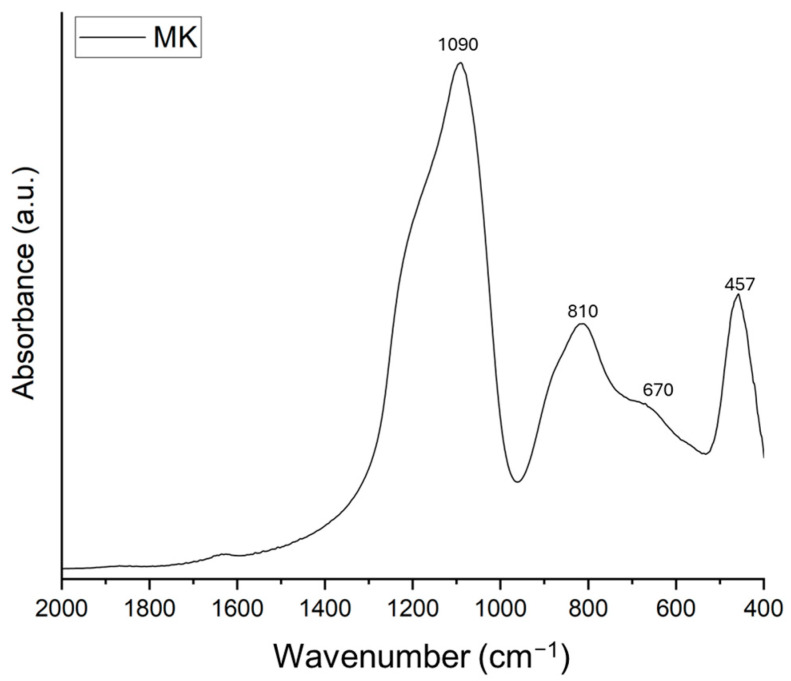
FT-IR spectra of MK.

The spectral analysis of MK is shown in Figure 4. The spectrum is characterized by four main absorption bands (1090, 810, 670, and 457 cm^−1^, respectively). The main absorption band at 1090 cm^−1^ is assigned to Si-O and Al-O stretching modes [35,37], while the band at 810 cm^−1^ is assigned to Al(VI)-OH or Al(VI)-O [35,38,39]. Finally, the bands at 670 and 457 cm^−1^ are ascribed to Al-O and Si-O bending modes [40].

**Figure 5:**
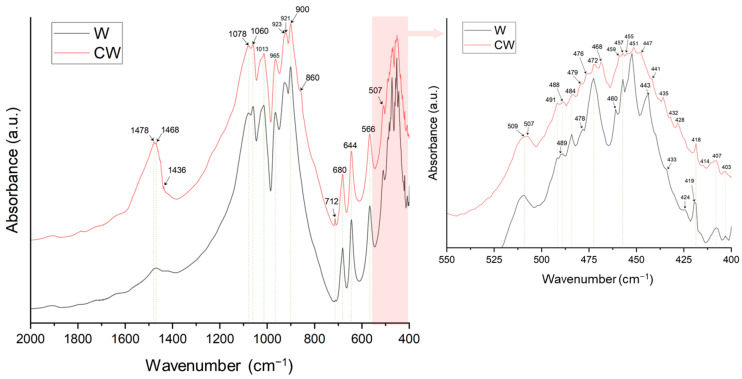
Comparison of FT-IR spectra of W and CW.

The IR spectra for W and CW are depicted in Figure 5. Both spectra reveal characteristic peaks of calcium silicate (CaSiO_3_), especially those in the 1100 to 850 cm^−1^ range and the 550 to 700 cm^−1^ range. Specifically, the peaks at 1078, 1060, and 1013 cm^−1^ are associated with the asymmetric stretching vibrations of Si-O-Si [29]. The peaks at 965 and 900 cm^−1^ correspond to the symmetric and asymmetric stretching vibrations of O-Si-O, respectively [41,42]. Peaks at 680 and 644 cm^−1^ are indicative of Si-O-Si symmetric stretching vibrations [33,43,44]. The bending vibrations of non-bridging and bridging oxygen in the Si-O groups are represented by peaks at 566, 507 and 472 cm^−1^ [45].

These peaks are present in both W and CW; however, noticeable differences emerge post-carbonation.

Firstly, the CW spectrum exhibits distinctive carbonate-related peaks, particularly those of calcium carbonate at 1478, 860, 712 and 468 cm^−1^ [46]. The first three peaks are attributed to the asymmetric stretching vibrations of the CO_3_^−2^ groups [47,48], while the last peak is assigned to Ca-O bending vibrations [49].

The second notable difference is seen in the decreased intensity of the silica-associated peaks in the CW sample compared to W. Specifically, some peaks in the 850 to 1100 cm^−1^ region and those between 600 and 400 cm^−1^ are less intense, suggesting that some of the silica may have been replaced or enveloped by carbonate groups during the carbonation process.

The differences observed in the carbonated and uncarbonated wollastonite are very important for the synthesis of the materials. Indeed, during carbonation, CO_2_ reacts with calcium from wollastonite to form calcium carbonate, which disrupts the crystalline structure and exposes more reactive sites. This increases the solubility of silicon and calcium, allowing these elements to more easily participate in the alkali activation process.

### 3.2. Samples Characterization

**Figure 6:**
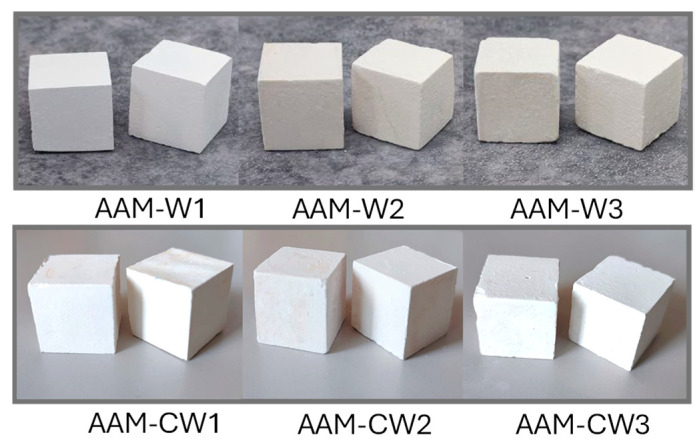
Samples appearance.

The produced samples are shown in Figure 6. Once removed from the molds, they appeared solid and bright white. Some of these, particularly AAM-W3 and AAM-CW3, left a white powdery residue upon handling, likely due to incomplete alkali activation. The samples AAM-W4 and AAM-CW4, once removed, were very fragile and could easily be broken under finger pressure, which led to their immediate rejection without further analysis.

**Figure 7:**
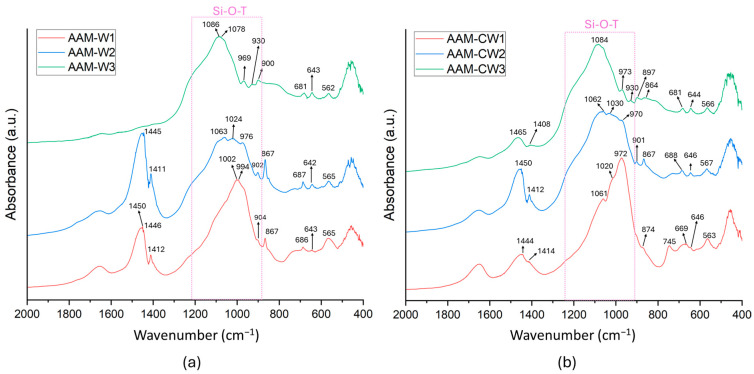
FT-IR analysis of AAM-W (**a**) and AAM-CW (**b**) samples.

Figure 7a,b show the IR spectra of the AAM-W and AAM-CW samples. As can be observed, all the samples exhibited characteristic bands indicating the formation of aluminosilicate networks. According to Chen et al. [50], the IR region between 920 and 1250 cm^−¹^ is associated with the asymmetric stretching of the Si-O-T bond (where T refers to Si or Al). The band in this region, typical of the N-A-S-H gel framework formation [51], can shift in wavenumbers depending on the Al/Si ratio and thus the quantity of Si-O-Si and Si-O-Al bonds present in the network. The Si-O-Si bonds have a higher bond strength compared to Si-O-Al bonds [52,53,54]; this results in higher wavenumber IR peaks for Si-O-Si bonds, while lower wavenumber peaks are associated with Si-O-Al bonds [50].

Comparing the samples AAM-W1, AAM-W2, and AAM-W3, as well as the samples AAM-CW1, AAM-CW2, and AAM-CW3, it is evident that the reduction of sodium hydroxide used in the various syntheses influenced the type of network formed.

Sample AAM-W1 was characterized by two peaks at 994 and 1002 cm^−¹^. As previously mentioned, these are attributable to the formation of Si-O-T bonds (where T = Si or Al). However, given that the peak was shifted towards lower wavenumbers, this means that there was a greater presence of Si-O-Al compared to Si-O-Si [50,55,56]. This is also evidenced by the peaks in the raw materials and the shoulder around 965 cm^−¹^, which is characteristic of O-Si-O bonds. A similar behavior was observed in AAM-CW1, where the peaks were recorded at 1061, 1020, and 972 cm^−¹^.

The AAM-W1 and AAM-CW1 samples were also characterized by peaks related to the O-C-O group. These peaks, which are found around 1400–1450 cm^−¹^, are attributed to the formation of calcium carbonate and the presence of C-A-S-H [57]. The formation of C-A-S-H in alkaline environments facilitates the presence of free calcium, which can react with atmospheric carbon dioxide to form carbonates [58]. Therefore, the presence of carbonates in the IR spectrum is a common indicator when C-A-S-H gels are formed, suggesting a reaction between the present calcium and environmental CO_2_.

Analyzing the AAM-W2 and AAM-CW2 samples, a prominent triple-peaked band is observable in the 900 to 1200 cm^−1^ region. The explanation for these distinct peaks, different from those in the other samples, is attributed to the intermediate amount of NaOH used compared to the other samples (AAM-W1/AAM-CW1 and AAM-W3/AAM-CW3). This amount might have been sufficient to activate a greater amount of silicon and aluminium than in AAM-W3 and AAM-CW3, but not so high as to cause excessive dissolution of the components as in AAM-W1 and AAM-CW1. Furthermore, the reduced NaOH content compared to the AAM-W1 and AAM-CW1 series allowed for a more balanced formation between N-A-S-H and C-A-S-H gels. In the AAM-W1 and AAM-CW1 systems, the high NaOH content could have lead to rapid solubilization of metakaolin and the formation of N-A-S-H species without allowing sufficient time for the calcium in the wollastonite to dissolve and thus form C-A-S-H. However, by reducing the NaOH quantity in the system, the formation of N-A-S-H could have been slowed, allowing for the dissolution of calcium from wollastonite and the formation of C-A-S-H. Additionally, it should be noted that the sodium in AAM-W2 and AAM-CW2 might not have been sufficient to balance all the negative charges of aluminium, which will therefore inevitably be balanced by calcium. Indeed, according to Hoyos- Montilla et Al., the co-presence of absorption bands at 1063–1062 cm^−1^ and 976–970 cm^−1^, and at 1030–1024 cm^−1^, are related to Si-O^−^ Na^+^ and Si-O^−^ Ca^2+^, which confirms the presence of both C-A-S-H and N-A-S-H gels in the consolidated AAM-W2 and AAM-CW2 materials [38].

The difference between AAM-W2 and AAM-CW2 was particularly evident in the intensity of the peaks, which were much more pronounced in AAM-CW2 than in AAM-W2. This difference is attributed to the carbonation of wollastonite, which has made the silicon and calcium more available for the reaction.

Finally, the AAM-W3 and AAM-CW3 samples appeared to be the least reacted. The peaks in the 900–1200 cm^−1^ range showed no significant variation compared to the raw materials. Additionally, the absence of the C-O peak in AAM-W3 indicated the absence of the C-A-S-H gel. Although a peak at 1465 cm^−1^ was present in AAM-CW3, it was much less intense compared to all other samples, suggesting in this case as well, a limited formation of the gel.

**Figure 8:**
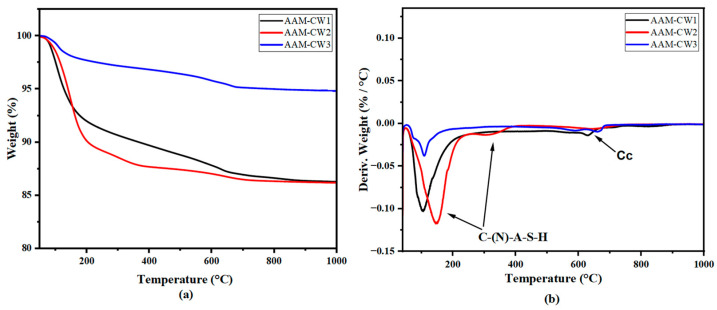
TGA curves of AAM-CW samples (**a**) and DTG of AAM-CW (**b**).

**Figure 9:**
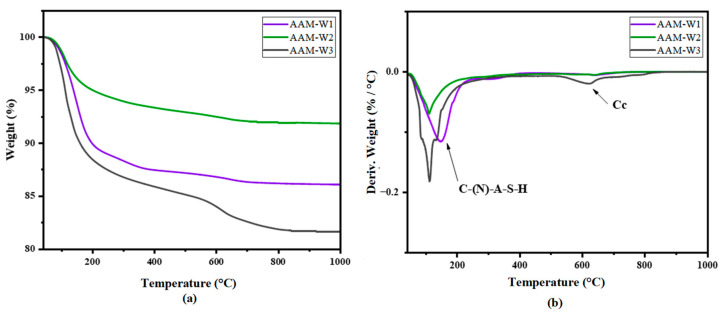
TGA curves of AAM-W samples (**a**) and DTG of AAM-CW (**b**).

Figure 8 and Figure 9 show the TGA curves of the samples. It can be seen that the main decomposition is in the range of 100–200 °C. The decomposition in this range in alkali-activated materials is ascribed to C-(N)-A-S-H gel formed as a product of geopolymerization. This gel product decomposition temperature is also reported by [59,60,61,62]. The C(N)ASH gel can be seen in AAM-CW2 and AAM-CW1 in relatively higher amounts compared to AAM-CW3 and AAM-W3. The TGA curves also reveal that AAM-CW2 contained a significant amount of C-(N)-A-S-H gel compared with AAM-CW1 and AAM-CW3. The distinct DTG curve of AAM-CW3 indicates dehydration of the material and thus implies a poorly formed or negligible gel as shown by the mass loss curve in Figure 8a. The sharp peaks in the thermograms of AAM-W3 and AAM-CW3 are likely caused by the dehydration of chemically bound water within micro-gels in the sample. These micro-gels may be concentrated in specific areas where more water was available, allowing limited gel formation, though not enough to form uniformly throughout the entire sample. AAM-CW2 showed a mass loss in the region of 200–400 °C, which probably indicates the presence of an anhydrous end member of C-(NASH) gel [61]. The mass loss in the 600–800 °C region was due to the decomposition of calcium carbonate in the carbonated samples [63]. The low mass loss shows that carbonation was low. The TGA results suggest that it is possible to form C-(N)-A-S-H gels through the alkali activation of wollastonite. However, when wollastonite is carbonated before alkali activation, higher amounts of C-(N)-A-S-H may form in samples with low carbonation compared to those with high carbonation. This is evidenced by the fact that, despite having a lower degree of carbonation, AAM-CW1 showed better carbonation than AAM-CW2, but contained less C-(N)-A-S-H gel than AAM-CW2. The carbonation of wollastonite implies that calcite must be leached to provide more calcium for C-(N)-A-S-H formation, which occurs slowly in high pH systems. It is also possible that the calcite products hinder the leaching of calcium from wollastonite by covering the precursor. Similar observations are seen in Figure 9a,b, in which the amorphous material identified in AAM-W3 and AAM-W2 in the TGA can be ascribed to the loss of water in the material.

**Figure 10:**
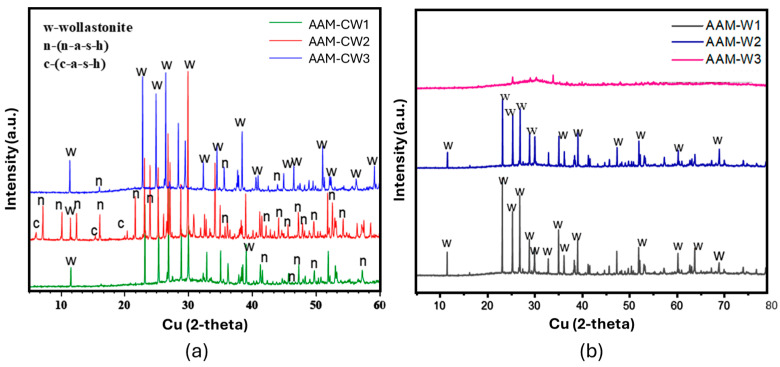
XRD results of AAM-CW samples (**a**) and AAM-W (**b**).

The presence of these products is also confirmed with XRD analysis, as shown in Figure 10a,b [64]. It can be observed that the AAM-W3 was mostly amorphous, confirming that it contained mostly the metakaolin precursor. It follows that in this case, the reactivity was poor, probably because of the unfavourable pH conditions.

## 4. Conclusions

This study explored the potential of alkali activation of metakaolin and wollastonite to create eco-friendly cementitious materials. The results showed that wollastonite can reduce the amount of sodium hydroxide required for the activation process. Remarkably, even with reduced sodium hydroxide and the absence of sodium silicate, the samples remained solid and successfully formed N-A-S-H and C-A-S-H gels, as confirmed by FT-IR, TGA, and XRD analyses.

The best-performing sample, AAM-CW2, demonstrated an optimal mix design, indicating that in this system, sodium hydroxide should not be reduced by more than 50%, and that the Na_2_O/Al_2_O_3_ ratio cannot be reduced below 0.5 without compromising the formation of both N-A-S-H and C-A-S-H gels. Indeed, maintaining high alkaline pH is necessary for occurrence of chemical reactions. In particular, samples AAM-W3 and AAM-CW3, which had insufficient sodium hydroxide, exhibited incomplete gel formation and weaker material properties.

The study also found that carbonation slightly improved reactivity by increasing the availability of silicon and calcium, which are fundamental for the formation of both N-A-S-H and C-A-S-H gels. The formation of gels was indeed more pronounced in AAM-CW2 than in AAM-W2.

Although this is a preliminary study, the results suggest that wollastonite-carbonated AAMs have the potential to serve as sustainable alternatives to traditional cement in certain construction applications. Potential uses may include building materials such as tiles, blocks, or precast elements, where low-carbon materials and environmental impact are key considerations. However, since mechanical performance testing and long-term durability studies were not conducted in this research, further investigation will be necessary to fully assess their suitability for broader applications, such as infrastructure projects like roadways, bridges, and foundations.

Future research could explore alternative methods to further enhance the availability of silicon and calcium in wollastonite, such as investigating acid activation as a potential alternative to alkaline activation. This approach could provide new avenues for optimizing the reactivity of wollastonite and reducing dependence on sodium-based activators, thereby improving the overall environmental footprint of these materials.

## Figures and Tables

**Table 1:** Composition of samples.

Label	MK (g)	W (g)	H_2_O (g)	NaOH (g)	SiO_2_/Al_2_O_3_	Na_2_O/Al_2_O_3_
AAM-W1	100.00	53.28	94.08	34.88	3	1.00
AAM-W2	100.00	53.28	85.36	17.44	3	0.50
AAM-W3	100.00	53.28	81.00	8.72	3	0.25
AAM-W4	100.00	53.28	76.84	0.40	3	0.01
AAM-CW1	100.00	53.28	94.08	34.88	3	1.00
AAM-CW2	100.00	53.28	85.36	17.44	3	0.50
AAM-CW3	100.00	53.28	81.00	8.72	3	0.25
AAM-CW4	100.00	53.28	76.84	0.40	3	0.01

## Data Availability

The original contributions presented in the study are included in the article, further inquiries can be directed to the corresponding authors.
